# Nordic treatment practices survey and consensus for treatment of eyelid sebaceous carcinoma

**DOI:** 10.1186/s12886-020-01367-3

**Published:** 2020-03-16

**Authors:** Tiina Leivo, Johanna Sarmela, Maria Enckell-Aaltonen, Eva Dafgård Kopp, Caroline Schmitt, Peter B. Toft, Haraldur Sigurdsson, Marita Uusitalo

**Affiliations:** 1 Department of Ophthalmology, Helsinki University Hospital and University of Helsinki, PO Box 220, FI-00029 HUS Helsinki, Finland; 2 Department of Ophthalmology, S:t Erik’s Eye Hospital, Karolinska Institutet, Stockholm, Sweden; 3 Department of Ophthalmology, Oslo University Hospital, Oslo, Norway; 4 Department of Ophthalmology, Rigshospitalet, University of Copenhagen, Copenhagen, Denmark; 5 Department of Ophthalmology, Landspitali, University of Iceland, Reykjavik, Iceland

**Keywords:** Sebaceous carcinoma, Eyelid, Treatment, Nordic, Consensus

## Abstract

**Background:**

The purpose was to describe the Nordic treatment practices and to reach a Nordic consensus for the treatment of sebaceous eyelid carcinoma.

**Methods:**

The treatment practices data was collected by a questionnaire with 37 questions to the Nordic oculoplastic surgeons and analyzed. A PubMed MEDLINE database search was done to gather data on the published treatment practices and recommendations. A working group that consisted of in minimum one senior consultant from each leading Nordic University Eye Hospital was assigned. A structured interactive method was used to establish the consensus.

**Results:**

Twenty-four doctors responded to the questionnaire. 23/24 (96%) of the respondents took a biopsy before surgery. Regional lymph node scanning was routinely done by 14/23 (61%) and a systemic screening of a metastatic disease by 13/23 (57%). 6/22 (27%) never took conjunctival mapping biopsies and 12/23 (52%) never screened for Muir- Torre. Respondents used Mohs surgery, frozen section or multi-stage excision with delayed closure, and 5–6 mm was the mostly preferred margin. Sentinel lymph node biopsy was a possible option for 9/22 (41%) and cryotherapy and Mitomycin C for 6/22 (27%) respondents. 50% of respondents considered radiation as a treatment option. 15/16 (94%) respondents always followed-up their patients, most for 5 years. Two thirds scanned regional lymph nodes during the follow-up. Consensus was reached for 18 statements representing three domains: preoperative work-up, treatment and follow-up.

**Conclusion:**

Treatment practices differ in between the five Nordic countries which have similar public health care systems. In the article the authors present a Nordic consensus for the treatment of eyelid sebaceous carcinoma.

## Background

Ocular sebaceous carcinoma is a rare malignant tumour, associated with significant morbidity and mortality. The tumour is rare in Latin American, Western and Nordic countries, i.e. the estimated annual incidence is 0.41 per million population in UK, but more common in Asian countries [1–4]. The median age of patients is 70 to 72 years [1, 5]. The relative population-matched 10-year survival is 79.2% [5]. Sebaceous carcinoma is often misdiagnosed clinically in patients with a history of a chronic unilateral conjunctivitis, blepharitis or a recurrent chalazion [1, 6, 7], and the delay has major adverse effect on patient mortality [6]. The upper eyelid is most often involved, followed by lower eyelid, palpebral conjunctiva and caruncle [1, 8]. The tumour is originated from Meibomian glands, or glands of Zeis and Moll. It shows two forms of growth; nodular and more diffuse epithelial form, the latter being more challenging to diagnose and treat [1, 6]. Some sebaceous tumours are associated with visceral malignancies, a rare inherited condition called Muir-Torre [8, 9].

The Nordic countries, Denmark, Finland, Iceland, Norway, and Sweden, have similar societies and health care systems, and relatively small populations. All the Nordic countries have a public health care system and strong emphasis on evidence-based medicine and cost-effective use of health care resources. To achieve these goals national guidelines are common for a vast scope of diseases. This forms an option for common Nordic treatment guidelines for a rare disease, like eyelid sebaceous carcinoma. There is no data on the current treatment- and follow-up practices of eyelid sebaceous carcinoma in the Nordic countries. Furthermore, guidelines do not exist but the literature contains guidance from the experience earned over the last 40 years by different research groups [6, 10–15].

The first aim of this study is to describe the Nordic treatment and follow-up practices of sebaceous eyelid carcinoma in relation to the published data. The second aim is to reach Nordic consensus for preoperative work-up, treatment and follow-up of sebaceous eyelid carcinoma.

## Methods

A questionnaire with 37 questions was designed with SurveyMonkey®. The questionnaire was divided into four sections. The first dealt with the background information, e.g. the country and the hospital of the respondent and the number of patients treated for eyelid sebaceous carcinoma both personally and at the hospital. The second dealt with the preoperative work-up, e.g. biopsy of the tumour, conjunctival mapping biopsies and screening for systemic disease and Muir-Torre. The third covered the treatment, e.g. the surgery (frozen sections, multi stage resection with delayed closure, Mohs surgery, aimed clinical margin, and exenteration), sentinel lymph node biopsy (SLNB), cryotherapy, postoperative radiation and Mitomycin C. The fourth focused on the clinical follow-up and the use of screening for systemic disease. The respondents remained anonymous.

The link to the questionnaire was sent by an email and a following reminder e-mail to ophthalmologists on the NOSOPRS (Nordic Society for Oculoplastic and Reconstructive Surgery) contact mailing list. It was also sent by a letter to the Head of all Ophthalmology Departments in the five Nordic Countries asking them to forward it to their doctors involved in oculoplastics. Additionally, the NOSOPRS contact persons in each Nordic Country were personally contacted to deliver the questionnaire. The responses were analyzed and presented by numerical and graphical proportions and percentages. If the number of responses was less than 24 in a question, the actual number of responses was shown in the denominator. Preliminary questionnaire results were presented and discussed at the NOSOPRS meeting in Finland in 2015.

PubMed MEDLINE database was searched for the terms “sebaceous carcinoma” and “eyelid” until February 2019. Publications written in English and reports of at least four patients and reviews were reviewed and statements regarding work-up, treatment and follow-up were collected. Cancer Staging refers to AJCC 8-th edition or 7-th edition. In AJCC 7-th T1 is <5 mm, not invading the tarsal plate or eyelid margin, in AJCC 8-th T1 is <10 mm. In AJCC 7-th T2a is > 5 mm but <10 mm or invades the tarsal plate or eyelid margin, T2b is 10 mm but <20 mm or involves the full thickness of the eyelid. In AJCC8-th T2 is > 10 mm but < 20 mm, T2a does not invade the tarsal plate or eyelid margin, T2b invades the tarsal plate or eyelid margin, T2c involves the full thickness of the eyelid. In AJCC 7-th T3a is > 20 mm or invades adjacent ocular or orbital structures or has perineural invasion. T3b requires enucleation, exenteration or bone resection. In AJCC8-th T3 is > 20 mm but < 30 mm, abc grading is equal to stage T2. In AJCC7-th T4 is not resectable, in AJCC 8-th T4 is any eyelid tumor that invades adjacent ocular, orbital or facial structures [16, 17].

A working group was assigned that consisted of one senior consultant, who treats eyelid sebaceous carcinoma patients, from each leading University Eye Hospital in Sweden, Norway, Denmark and Iceland and the original Finnish working group of four senior consultants. A structured method was used to reach the consensus. Eighteen structured questions were formulated and subsequently divided between authors TL, JS, MEA, EDK and MU. In three interactive telephone meetings all questions were dealt with a similar protocol. In round 1 the designated author of the question asked the structured question and presented the applicable results of the literature review. Every author expressed her opinion on the question in her turn. Areas of disagreement where discussed in the interactive meeting. If consensus was reached, the statement was formulated in the meeting. In round 2 questions that did not reach consensus in the meetings were further formulated and discussed in open e-mails with all authors until a final statement was reached. If a categorical agreement was not reached the minimum level of jointly agreed treatment practice was recommended or a conditional formulation was used. Areas of disagreement were specified in the consensus.

All authors contributed substantially to the present study. The study has followed the Tenets of Declaration of Helsinki.

## Results

Twenty-four doctors responded to the questionnaire, eight from each Finland and Sweden, six from Norway, one from Denmark, and one from Iceland. As doctor in charge of the treatment 19 respondents had treated 1–10 sebaceous carcinoma patients in total during their carriers, 2 respondents 11–20 patients and one doctor over 20 patients. Two doctors did not answer the question.

### Preoperative work-up

**Fig. 1:**
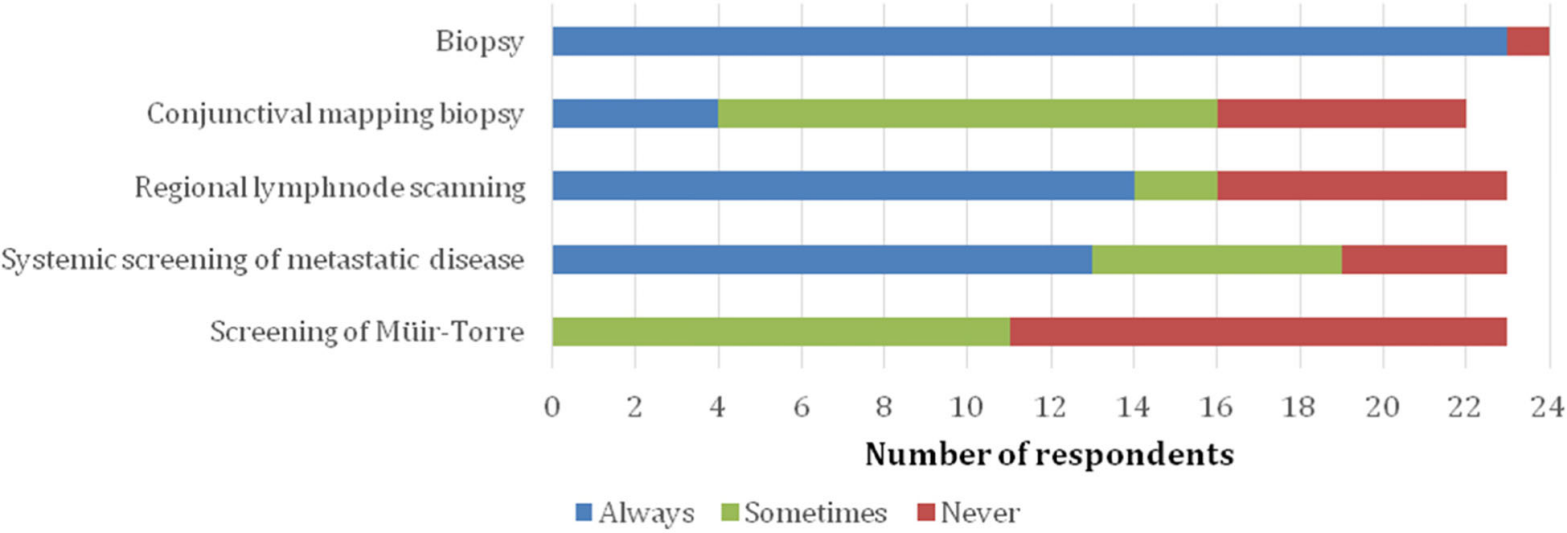
Preoperative work-up used for sebaceous eyelid carcinoma in the Nordic countries

23/24 (96%) of the respondents took a biopsy of a suspected lesion before surgery. Punch biopsy was taken by 14/24 (58%) and an incisional wedge biopsy by 10/24 (42%). Conjunctival mapping biopsies were routinely taken by 4/22 (18%) of the respondents. 12/22 (55%) took conjunctival biopsies when the tumour was large, clinical signs of pagetoid disease existed, conjunctiva looked irritated or the lesion involved the eyelid margin. 9/22 (41%) took conjunctival biopsies before surgery and 5/24 (21%) at the time of surgery (Fig. 1).

Regional lymph node scanning was routinely done by 14/23 (61%) and a systemic work-up of a metastatic disease by 13/23 (57%). The reasons for occasional work-up (6/23, 26% of respondents) were the suspicion of pagetoid disease or if an exenteration was planned. Lungs and liver were screened by 12/23 (52%).

Screening, in any form, for Muir-Torre demonstrated a clear dispersion between the Nordic countries. None of the respondents routinely screened for Muir-Torre, but in Sweden 6/8 (75%) of the respondents sometimes screened. In the rest of the Nordic countries screening for Muir-Torre was rare. From all respondents 12/23 (52%) never screened for Muir- Torre. The reasons for occasionally screening were positive family cancer history, multiple malignant tumours and the patient’s request for screening.

### Treatment

**Fig. 2:**
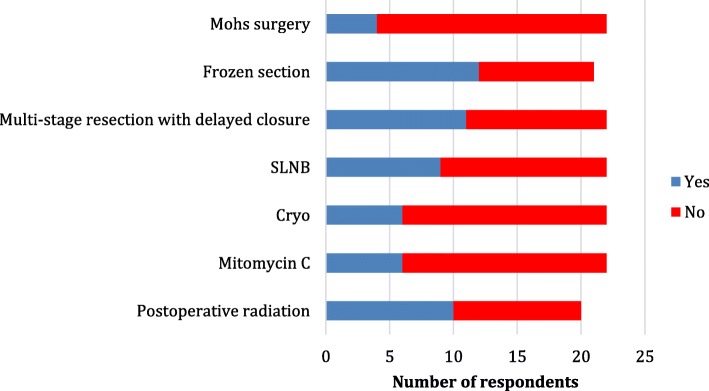
Treatment modalities used for sebaceous eyelid carcinoma in the Nordic countries

**Fig. 3:**
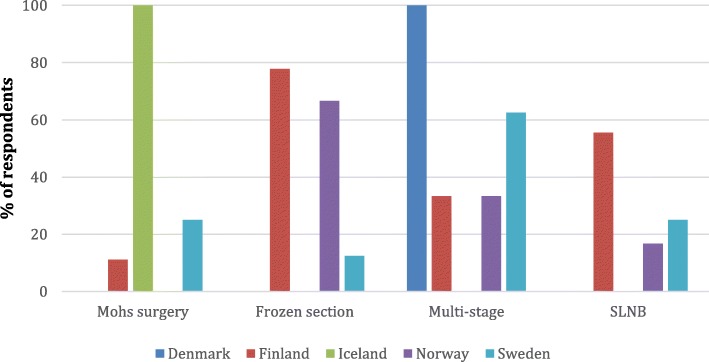
Treatment modalities used by Nordic country for verifying margins

Figures 2 and 3 demonstrate the various treatment modalities used. Mohs surgery was the preferred method in Iceland, frozen section are used especially in Norway and Finland and multi-stage resection in Sweden and Denmark. Sentinel lymph node biopsy was used by 9/22 (41%).

Cryotherapy and Mitomycin C is a possible adjuvant treatment option for 6/22 (27%) respondents. Mitomycin C was used only in Sweden and Finland. Postoperative radiation is considered a treatment option for 10/20 (50%) respondents, and mainly when margins are considered insufficient. The aimed clinical margin at surgery was 3–4 mm for 8/20 (40%), 5–6 mm for 9/20 (45%) and in minimum 7 mm for 3/20 (15%) respondents.

The rate of exenteration was low, only 5/19 (26%) had done an exenteration due to extensive disease during the last 15 years. 12/20 (60%) would though consider exenteration in cases with extensive conjunctival disease or local orbital disease. 18/20 (90%) would consider exenteration if extensive orbital disease.

### Follow-up

15/16 (94%) respondents always follow-up their patient, one respondent does not follow-up if the tumor is small and a 4–5 mm margin is obtained. Eight respondents did not answer the question.

The length of the follow-up was specified by 13 respondents. 11/13 (85%) respondents followed their patient for 5 years and two for 2–3 years. The frequency of follow-up was variable; every 2–6 months first 1–3 years and every 6–12 months last 2–5 years. No one followed-up their patients for more than 5 years.

**Fig. 4:**
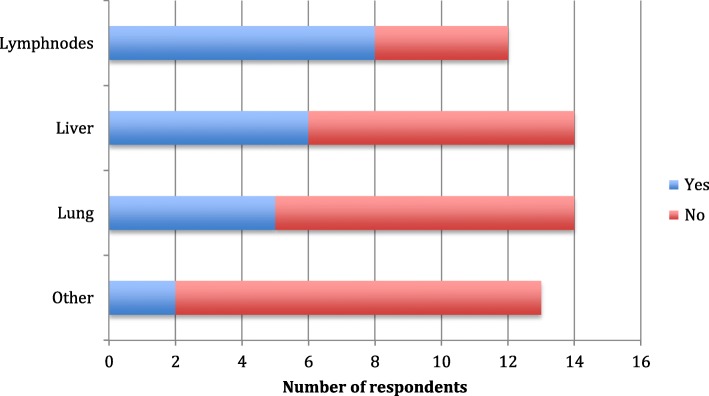
Scanning for regional lymph nodes or metastatic disease during follow-up in the Nordic countries

Scanning by CT, MRI or ultrasound for metastatic disease during follow-up is presented in Fig. 4. Most respondents scan the regional lymph nodes as often and as long as they perform the clinical follow-up. In Denmark no annual scanning for regional lymph nodes or metastatic disease was used.

## Discussion

There was only one respondent from Denmark, and one from Iceland. We consider the results still relevant as the treatment of eyelid sebaceous carcinoma is centralized to only two centers in Denmark and one center in Iceland. In the other three Nordic countries many centers treat this extremely rare disease. Due to its rarity centralization could be considered in all Nordic countries.

### Preoperative work-up

Preoperatively, a comprehensive clinical evaluation including examination of the eye, conjunctiva and caruncle is recommended. All eyelids should be everted and preauricular, submandibular, parotic and cervical lymph nodes should be palpated [8, 13].

To establish the diagnosis a full thickness eyelid biopsy including skin, tarsus and palpebral conjunctiva is recommended by Shields et al. [13] This can be done by an incisional biopsy or with a round penetrating trephine. According to Shields et al. [13] chest x-ray, liver enzyme blood tests and work-up by MRI or CT are not necessary in small lesions. However, if there is a suspicion of orbital invasion or if the risk of nodal metastasis is high, T2b or more (AJCC 7-th edition), a orbital CT or MRI, thorax CT, parotid, submandibular and cervical lymph nodes ultrasonography or CT and possible fine needle aspiration biopsy are needed [8, 13, 18].

Conjunctival mapping biopsies are recommended to ensure tumour margins as sebaceous carcinoma has a tendency of intraepithelial spread. They are recommended if there is suspicion of conjunctival or caruncular involvement [1, 13, 19, 20]. Shields et al. [13] report that routinely four biopsies from the palpebral and six from the bulbar conjunctiva should be taken. If corneal involvement is suspected, 4 additional biopsies close to limbus should be taken. Permanent sections are recommended [13]. A recent publication by McConnell indicates that the pattern and location of the primary tumour do not correlate to a possible intraepithelial spread and therefore standardized mapping biopsies are always recommended [21]. Contrary to these recommendations, in our study 27% of respondents never used and 54 % occasionally used conjunctival mapping biopsies.

Muir-Torre syndrome (MTS) is a rare inherited condition characterized by a combination of sebaceous carcinoma, other sebaceous gland tumors and other malignant tumours; colorectal, genitourinary and breast cancer being the most common [1, 8, 9]. The opinions regarding screening for MTS vary, but it is suggested to rule out visceral malignancies as 6–20% of individuals with periocular sebaceous carcinoma develop visceral tumours [18, 22]. In one series with eyelid sebaceous cancer, MTS was found in 1 of 34 patients [1], however, in another series, 10 of 31 patients had MTS as indicated by clinical diagnosis of visceral malignancies [23]. History of multiple sebaceous neoplasms, other tumours connected to Lynch syndrome (hereditary nonpolyposis colorectal cancer) in the patient or in a relative and age under 60 years are risk factors for MTS [24]. It is notable that in our study 52% of respondents never screened for MTS, which indicates a need for reconsideration.

### Treatment

Full-thickness eyelid tumor resection with posterior lamellar is the golden standard and superior to non posterior lamellar resection [25, 26]. Mohs surgery has been recommended as treatment of sebaceous carcinoma with excellent results with 11% recurrence rate for primary tumours [27–31]. However, Mohs surgery is resources- and time-demanding and not available everywhere. Good results have also been published using frozen sections and therefore many authors prefer it [6, 8, 13, 20, 32, 33]. Some prefer multi-stage resection with permanent margins and delayed closure, reporting 12% recurrence rates [7, 34, 35]. Multicentricity of sebaceous carcinoma makes the securing of clear margins difficult with all methods. There are reports with less favorable results using both Mohs surgery [12, 36, 37] and frozen section [12]. In our study 57% used frozen section, 50% multi-stage resection with delayed closure and 18% Mohs surgery, i.e. some respondents had more than one strategy dependent on the case. The technique differed by countries, reflecting, presumably, the facilities available and the surgeon preference.

A 1–3 mm margin is reported to have a recurrence rate of 36% and a 5 mm margin a recurrence rate of 0% [10]. Good results have been reported using 4 mm [6, 38–40], 5 mm [13, 41, 42], as well as 5 to 6 mm margins [43]. It seems reasonable to aim for at least 4 mm margins. The majority of our respondents aimed for 5–6 mm margins.

Sentinel lymph node biopsy (SLNB) was used by 41% of all respondents and more commonly in Finland. Recent reports have recommended the use of SLNB in patients with eyelid sebaceous carcinoma with tumours T2b (AJCC 7-th edition) or worse and in recurrent tumours [15, 18, 44–48]. SLNB has been reported to be beneficial in skin melanoma and Merkel cell carcinoma, but SLNB has not been proven to increase survival in eyelid malignancies [49]. Pfeiffer et al. [50] have reported SLNB to identify nodal micrometastasis in 20% of ocular adnexal melanoma cases. However, in tumors, such as sebaceous carcinoma, that spread via the lymphatic system before spreading systematically SLNB is justifiable in tumors with a significant metastatic risk [30].

Shields et al. [13] strongly advise to use cryotherapy for every patient, both at time of conjunctival biopsy and final surgery [13, 14, 19]. However, in Esmaeli’s series of 50 patients, no patient was treated with cryotherapy, with similar outcome [46].

There are few publications with few patients regarding topical Mitomycin C treatment for sebaceous carcinoma of the conjunctiva. Still most authors recommend it as an alternative in treating residual in situ growth in the conjunctiva [6, 8, 46]. In cases with extensive conjunctival spread Shields et al. [51] and Xu et al. [49] recommend a combination of cryo and Mitomycin C. These options could always be considered as an alternative to large conjunctival resections and exenteration. However, their use was considered by only 27% of respondents, which is surprisingly low.

Postoperative radiation after exenteration in T3 tumors (AJCC 7-th edition) is reported to significantly reduce the risk of recurrent disease [52]. Radiation has also been recommended for recurrent disease after exenteration [13] and postoperatively if insufficient margins [46] or perineural spread [8, 30, 46] is detected. Xu et al. [49] recommend radiation therapy for T3 or higher (AJCC 7-th edition), pagetoid spread, nodal metastasis or palliative care.

Radiation as primary therapy for patients who are inoperable or refuse exenteration has shown surprisingly good results when the dose of radiation has been 50–60 Gy. In a series published by Hata [53], all 5 patients who got radiation as primary treatment, were alive after 5 years. Radiation might be underused in the Nordic countries as only 50% would consider postoperative radiation. However, the risk of radiation induced dry eye, radiation keratopathy, radiation retinopathy, cataract and even a painful blind eye and secondary tumors should be kept in mind.

Few of our respondents have ever performed an exenteration in sebaceous carcinoma patients. In older published series the rate of exenteration was around 10% [1, 6, 7]. Today it can often be avoided with adjuvant treatments, such as local mitomycin C and cryotherapy in cases with conjunctival spread [53]. Nevertheless, 60% of our respondents would still consider exenteration in these cases. Exenteration should be considered if there is extensive growth in the orbit or recurrent orbital disease after globe sparing surgery. A very interesting recent study [54] demonstrated that preoperative chemoterapy reduced tumour size remarkably and spared the patients from exenteration. The follow-up was only 18 months, but chemoreduction seems probably a promising treatment method [55].

The use of PET/CT was not included in the survey, and none of the respondents reported using PET/CT in the open questions. There is little published data on the role of PET/ CT for staging and treatment of sebaceous eyelid carcinoma, but published data support e.g. selected use of PET/CT for the management of head and neck squamous cell cancer [56]. Selected use of PET/CT could also be considered in the management of sebaceous eyelid carcinoma.

### Follow-up

The length of follow-up is debatable. Many studies report 5 years or longer follow-up. It is noteworthy that local recurrences or metastases have been reported after 60 months [7], 71 months [41] and 132 months [57] in patients who have had free margins. The reported median times from initial treatment to recurrence are 16.5 to 25 months. The reported rates for nodal recurrences are 8–23% and for distant metastasis 2–14% [6–8, 11, 26, 46, 58, 59].

A higher risk for recurrence or metastasis has been reported in patients with symptoms over 6 months, involvement of both upper and lower eyelids, multicentric origin, diffuse or a non-lobular pattern, pagetoid spread, orbital involvement, perineural invasion and stage T2b (AJCC 7th -edition) or worse [60, 61]. In a chinese study of 238 patients, risk factors for tumor-related death were orbital involvement, the greatest tumor basal diameter, pagetoid spread and lymph node metastasis at initial diagnosis [62]. From United States Sa et al. [15] report in study of 100 patients, that T3b or worse (AJCC 8-th edition) and N1 are risk factors for death, Lee et al. [63] report in study of 940 patients, that older age and greater tumor size correlate with decreased overall survival, whereas surgical treatment or combined surgical and radiation treatment correlate with increased overall survival.

Stage T2b (AJCC 7th-edition) and stage T2c (AJCC 8-th edition) or a more advanced disease is reported to correlate with regional lymph node metastasis and T2b or T3a or worse (AJCC 7th-edition) and stage T2c (AJCC 8-th edition) are reported to correlate with distant metastases [11, 15, 46, 58]. Kaliki et al. [61] estimate of lymph node metastasis at 5 and 10 years are 0 and 0% for T1, 11and 11% for T2, 44 and 59% for T3 and 100 and 100% for T4, which would justify a longer than 5 year follow-up for T3 (AJCC 7th- edition) or worse.

Few studies include recommendations for follow-up, but some authors emphasize its importance [7, 8, 44]. Yin et al. [8] recommend a minimum of 5 years of follow-up, including clinical examination. Patients with an increased risk of nodal metastases are recommended to have a clinical examination for lymph nodes and a neck ultrasonography. Further, Yin et al. [8] recommend imaging of the orbit for exenterated patients and possible lung scans in advanced cases. For T2b or worse (AJCC 7-th edition) or T2c or worse (AJCC 8-th edition) a strict regional lymph node surveillance is recommended by Esmaeli et al. [46], Choi et al. [58], Lam et al. [48] and Sa et al. [15], furthermore a surveillance for distant metastases is recommended by Choi et al. [58] and Sa et al. [15].

In our study most respondents used a 5-year follow-up, which in the light of published studies seems a justified minimum follow-up time. However, all respondents did not follow-up their patient, which needs reconsideration.

## Conclusion

Treatment practices differ in between the five Nordic countries. Here we present a Nordic consensus for the work-up, treatment and follow-up of eyelid sebaceous carcinoma (Table 1).

**Table 1:** Nordic consensus for treating sebaceous carcinoma of the eyelid

Practice	Statement	References
**Preoperative work-up**		
Biopsy	A full thickness or in minimum incisional biopsy, request histological analysis for sebaceous cancer.	[13]
Biopsy in chalazion surgery	Request histological analysis, when the lesion is clinically suspicious or recurrent.	[13]
Preoperative conjunctival mapping biopsies	Consider, if there is suspected conjunctival involvement.	[1, 13, 19–21]
Regional lymph node scanning	Offer for category T2b (AJCC 7-th edition) or T2c (AJCC 8th- edition) and worse.	[8, 13, 18]
Colonoscopy	Should preferably be offered for all patients with sebaceous cancer. 1)	[18, 22]
A genetic counseling for Muir-Torre syndrome	Should preferably be offered if: • two or more primary sebaceous tumours in one patient and/or • under 60 years old and history of another MTS or Lynch cancer (colon, rectum, endometrial, ovarian, small bowel, gastric, urinary tract and brain) and/or • under 60 years old and at least one first degree relative with a tumour above. 2)	[24]
**Treatment**		
Primary treatment method	Surgery with posterior lamellar resection.	[25, 26]
Clinical margin	At least 4–5 mm. 3)	[6, 10, 13, 38–43]
Method of surgery	Multi-stage resection with delayed closure, frozen sections or Mohs surgery are recommended to verify tumour-free margins. Conjunctival mapping biopsies can be performed together with the final surgery if performed as multi-stage resection with delayed closure.	[6–8, 12, 13, 20, 27–37]
Sentinel lymph node biopsies	SLNB could be considered for tumours larger than 10 mm.	[15, 18, 30, 44–50]
PET/CT	PET/CT could be considered in the initial staging.	[56]
Cryo	In cases with pagetoid spread, additional cryotherapy to the remaining conjunctiva is optional. The primary treatment is local resection if possible without extensive conjunctival resection.	[13, 14, 19, 46, 53]
Mitomycin-C	In cases with extensive conjunctival epithelial spread or residual conjunctival disease, topical Mitomycin- C could be considered as an alternative to extensive surgery or exenteration. If there is growth deep to the epithelium, Mitomycin-C is not an option.	[6, 8, 46, 49, 51]
Postoperative adjuvant radiation	Offer radiation for tumors staged T3 (AJCC 7-th edition) or more and in cases with perineural spread or insufficient margins. For patients who deny surgery, radiation at a sufficient dose could be considered.	[8, 13, 30, 46, 49, 52, 53]
Preoperative chemoreduction	In selected cases preoperative chemoreduction can be considered.	[54, 55]
**Follow-up**		
The length of the follow-up	In minimum 5 years. 4)	[6–8, 11, 26, 41, 46, 57–59, 61]
Clinical follow-up interval	Follow-up interval is individual and depends on the post-diagnosis time-frame. In most cases four to 6 months follow-up interval can be considered.	–
Follow-up examinations	The follow-up should in minimum comprise a clinical examination and palpation for lymph nodes. Patients should also be instructed to palpate the lymph nodes themselves in-between follow-ups. Annual scanning (ultrasound or MRI) for regional lymph node metastases is recommended. Scanning for distant metastases could be considered for category T2b (AJCC 7-th edition) or T2c (AJCC 8-th edition) or worse.	[8, 15, 46, 48, 58]

Areas of disagreement

1) Some authors categorically recommended colonoscopy

2) Some authors categorically recommended Muir - Torre screening in the above defined cases

3) Some authors recommended a minimum margin of 4 mm and some 5 mm

4) Some authors recommended a follow-up period of 10 years

## Supplementary information

**Additional file 1.** Questionnaire questions.

## Data Availability

The Nordic oculoplastic surgeons’ survey data is not available, as it may comprise the individual data privacy of the respondens, as some of them might be identified.

## Abbreviations

AJCC: American Joint Committee on Cancer
MTS: Muir-Torre syndrome
NOSOPRS: Nordic Society for Oculoplastic and Reconstructive Surgery
SLNB: Sentinel lymph node biopsy
